# Capsule Endoscopy in a Patient with an Implanted CCM System and an Implantable Defibrillator

**DOI:** 10.1155/2011/275659

**Published:** 2011-07-27

**Authors:** Florian Streitner, Nina Schoene, Martin Borggrefe, Jürgen Kuschyk

**Affiliations:** 1st Department of Medicine-Cardiology, University Medical Centre Mannheim, 68167 Mannheim, Germany

## Abstract

Wireless video capsule endoscopy (CE) is a modern diagnostic tool. Because of its use of digital radiofrequency, it is still relatively contraindicated in patients with implanted cardiac devices. We report the case of a patient with an Optimizer III system delivering cardiac contractility modulating signals (CCM) for heart failure therapy and an implantable cardioverter defibrillator (ICD) who underwent CE. No interferences between the devices were found.

## 1. Introduction

Wireless video capsule endoscopy (CE) has become the gold standard in patients with unknown blood loss, particularly when there is a high suspicion of small bowel disease [1]. Images acquired by CE are continuously transmitted by radiofrequency converted into electromagnetic waves to the recorder unit located around the patients waist. Studies to date suggest that CE is associated with few adverse events [2, 3], but the presence of implanted cardiac devices still represents a relative contraindication for several external electromagnetic source applications because of potential interference causing transient or permanent device dysfunction [4]. Therapy with implantable cardioverter-defibrillators (ICDs) itself is well established, whereas cardiac contractility modulation (CCM) represents an upcoming new device therapy developed for heart failure treatment with increasing numbers of devices being implanted worldwide. A CCM device works by stimulating the right ventricular septum with two screw-in leads. The device delivers high-energy stimulation during the absolute refractory period of the heart, thus improving cardiac contractility by influencing calcium fluxes [5]. 

We report a case of a patient with a CCM System (OPTIMIZER III, Impulse Dynamics USA, Inc., Orangeburg, NY) and a dual-chamber ICD (Atlas + DR V-243, St. Jude Medical, Inc., Sunnyvale, CA) in whom CE (Given PillCam SB, Yoqneam, Israel) was performed. Possible interferences between the three electronic devices were evaluated.

## 2. Case Report

A 74-year-old man was admitted to our hospital because of melena leading to a normochromic normocytic anaemia (haemoglobin on admission 7.5 g/dl, MCV 91 fl, MCH 30.7 pg, MCHC 33 g/dl). Oral anticoagulation due to mitral valve replacement and intermittent atrial fibrillation was temporarily discontinued. Oesophagogastroduodenoscopy and colonoscopy both gave negative results. As a consequence, CE was planned to examine the small bowel mucosa despite a concern regarding potential interferences between the radiofrequency emitted by the capsule and the ICD or the CCM device. The CCM device was programmed to deliver therapy 10 hours/day (1 hour CCM delivery, 1.4 hours discontinuation). 

**Figure 1:**
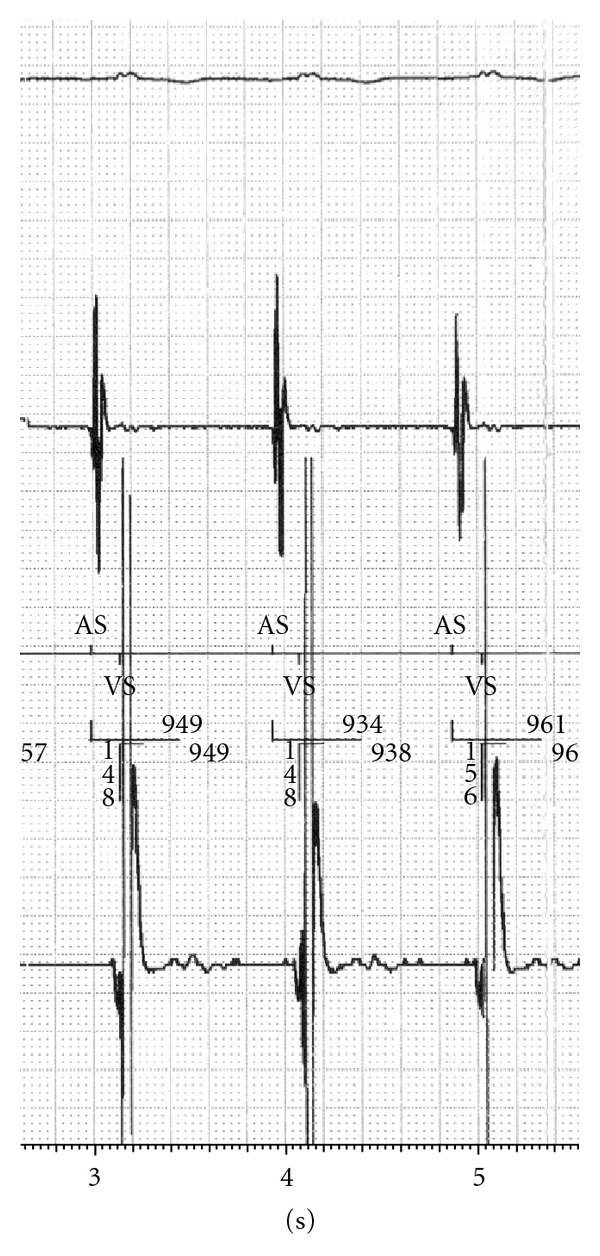
ICD Printout: CE, ICD, and CCM active.

**Figure 2:**
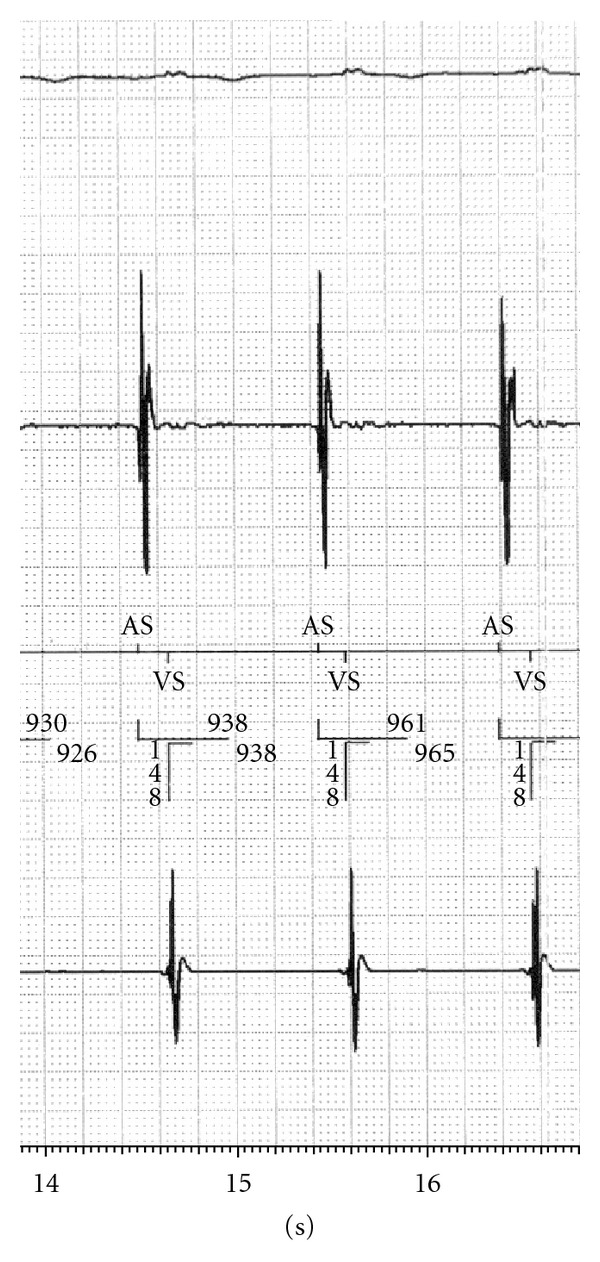
ICD Printout: CE and ICD active, CCM off.

After ingesting the PillCam SB capsule, the patient was admitted to the intensive care unit. The CCM and ICD device settings were left unchanged (Table 1). Continuous monitoring of ECG and vital signs was performed. The ICD as well as the CCM device were interrogated before, twice during, and directly after the CE procedure. Cross talk tests to exclude oversensing of the ICD leading to inadequate detection of ventricular tachyarrhythmia due to CE signal transmission or malfunction of the CCM device were performed. During CE alone or together with CCM delivery, continuous sinus rhythm with a high pacing percentage (80%) was noticed. No ventricular tachyarrhythmia or other cardiac adverse events were observed. No short-term artefacts due to CE radiofrequency transmission or due to altered CCM signal delivery were detected during visual online observation of the atrial and ventricular ICD channels (Figures 1 and 2) or during continuous ECG monitoring. No temporary discontinuation or total inactivation of the CCM device was noticed. Active CE did not influence the timing of the atrial triggered ventricular high energy stimulation leading to a safe CCM therapy delivery. Both active cardiac devices did not affect CE recordings regarding quality or quantity of the transmitted data. 

## 3. Discussion

By now, there are no reports regarding CE in patients with a CCM device, and only a limited number of studies have investigated the potential interference between CE and ICDs. Dubner et al. reported an inappropriate shock therapy during in vitro studies in one out of six different tested ICD devices due to electromagnetic interference and raised safety concerns regarding CE in patients with an ICD [6]. In general, radiofrequency emitted as electromagnetic waves could explain malfunctions of cardiac devices due to a very sensitive setting of special band-pass filters in their sensing circuits. However, the implanted CCM and ICD devices, both using frequencies in the range of kHz for communication, did not reveal any malfunctions during CE procedure. Additionally, picture transmission of the PillCam SB to the recorder, which is conducted with a 434.09 MHz carrier frequency and 2 Hz pulse trains (250 ms on, 250 ms off) was processed unimpaired leading to clear endoscopic images during the entire recording period. 

In summary, no device malfunctions in a patient with an CCM device and an ICD were observed during CE, but because of limited experience, the use of CE in these patients should only be performed in conjunction with close monitoring.

##  Conflict of Interests

The authors declare no conflict of interests. 

## Figures and Tables

**Table 1:** Device settings.

Optimizer settings	
Initiation time	00:00
Termination time	24:00
CCM (hours/day)	10
CCM signal delay (ms)	50
CCM signal amplitude (V)	6.0
CCM signal total duration (ms)	5.14
Sensitivity RA Lead (mV)	2
Sensitivity RV Lead (mV)	2
Sensitivity LS Lead (mV)	4.1
AV delay (ms)	252

**ICD settings**

Pacer programming (DDD/min)	60–130
VT detection zone (bpm)	>150
VF detection zone (bpm)	>231
PVARB (ms)	310
Ventricular refractory period (ms)	250
Sensitivity RA Lead (mV)	0.2 (auto)
Sensitivity RV Lead (mV)	0.3 (auto)
A/V post-sensed decay delay (ms)	0/60
A/V post-sensed threshold (%)	50/62.5

CCM: cardiac contractility modulation; RA: right atrium; RV: right ventricle; LS: lower septal; AV: atrioventricular; VT: ventricular tachycardia; VF: ventricular fibrillation; PVARB: postventricular atrial refractory period.
